# Covalent Organic Frameworks Composites Containing Bipyridine Metal Complex for Oxygen Evolution and Methane Conversion

**DOI:** 10.3390/molecules27165193

**Published:** 2022-08-15

**Authors:** Xin Liu, Lijuan Feng, Yongpeng Li, Tian Xia, Zhuyin Sui, Qi Chen

**Affiliations:** 1 Department of Bioengineering, Zhuhai Campus of Zunyi Medical University, Zhuhai 519041, China; 2 State Key Laboratory of Marine Resource Utilization in South China Sea, Hainan University, Haikou 570228, China; 3 School of Chemistry & Chemical Engineering, Yantai University, Yantai 264005, China

**Keywords:** covalent organic frameworks, multimetal, methane, oxygen evolution reaction, electrocatalyst

## Abstract

Novel covalent organic framework (COF) composites containing a bipyridine multimetal complex were designed and obtained via the coordination interaction between bipyridine groups and metal ions. The obtained Pt and polyoxometalate (POM)–loaded COF complex (POM–Pt@COF–TB) exhibited excellent oxidation of methane. In addition, the resultant Co/Fe–based COF composites achieved great performance in an electrocatalytic oxygen evolution reaction (OER). Compared with Co–modified COFs (Co@COF–TB), the optimized bimetallic modified COF composites (Co_0.75_Fe_0.25_@COF–TB) exhibited great performance for electrocatalytic OER activity, showing a lower overpotential of 331 mV at 10 mA cm^−2^. Meanwhile, Co_0.75_Fe_0.25_@COF–TB also possessed a great turnover frequency (TOF) value (0.119 s^−1^) at the overpotential of 330 mV, which exhibited high efficiency in the utilization of metal atoms and was better than that of many reported COF-based OER electrocatalysts. This work provides a new perspective for the future coordination of COFs with bimetallic or polymetallic ions, and broadens the application of COFs in methane conversion and electrocatalytic oxygen evolution.

## 1. Introduction

With the increasingly serious global energy shortage and environmental pollution, the exploration of green energy such as solar [1,2], hydrogen [3,4] and methane [5,6] energy has drawn much attention. Metal, as an important catalyst, has been widely studied and used in the production and efficient conversion of green energy sources. Specifically, precious metal materials such as ruthenium-based and iridium–based catalysts are widely used as efficient electrocatalysts for OER in order to improve the low efficiency of the anodic oxygen precipitation reaction (OER) caused by the slow kinetics during electron transfer [7,8,9]. Moreover, platinum–based catalysts exhibited excellent catalytic performance for the direct conversion of methane [10,11]. However, the scarcity, high cost, and secondary pollution to the environment have hampered the application of precious metals [12]. Therefore, it is increasingly important to reduce the use of precious metals, and develop cost-effective and efficient metal composite catalysts.

As an emerging family of organic porous polymers, covalent organic frameworks (COFs) were widely reported in adsorption [13,14,15,16], sensing [17,18,19,20], energy storage [21,22,23,24], optoelectronics [25,26,27,28], and catalysis [29,30,31]. Benefitting from their large surface area, and regular and adjustable open nanopores, COFs are suitable for doping with externally useful active metals to be used as a catalyst [12,32]. On the one hand, metal–loaded COF composite catalysts can effectively reduce the use of metals and improve the efficiency of atomic utilization. On the other hand, because of the low catalytic activity and poor electrical conductivity of primitive COFs, the introduction of metal can effectively improve the application of COF materials in catalysis, especially electrocatalysis [33,34]. However, many reports about COF–based catalysts mainly focused on monometallic modified COF composites. Compared with monometallic loaded COF composites, bimetallic loaded COF–based catalysts can effectively avoid the reduction in catalytic ability caused by the loss of metal species and filling of central vacancies during the catalytic process, which can improve the efficiency of atom utilization [35,36]. Therefore, the construction of efficient and cost–effective bimetallic or polymetallic loaded COF composite catalysts has become an important issue that needs to be further investigated.

In this work, we designed and prepared different covalent organic frameworks composites containing bipyridine metal complex. Benefitting from the coordination interactions between the N atom in bipyridine groups and the metal ions, the POM–Pt@COF–TB and a series of Co/Fe–based COF composites were obtained. Owing to the presence of active Pt sites, POM–Pt@COF–TB displayed catalytic performance for the conversion of methane. After the introduction of different amounts of Fe^3+^ and Co^2+^, Co_0.75_Fe_0.25_@COF–TB exhibited better performance for oxygen evolution reaction than that of other Co/Fe–based COF composites. The overpotential of Co_0.75_Fe_0.25_@COF–TB (331 mV, 10 mA cm^−2^) was superior to that of other composites. Meanwhile, its catalytic performance remained great after 1000 cycles. This work provides a new perspective for the synthesis of COF composites containing a bipyridine multimetal complex, and broadens the design of catalysts for methane conversion and electrocatalytic OER.

## 2. Results and Discussion

**Scheme 1:**
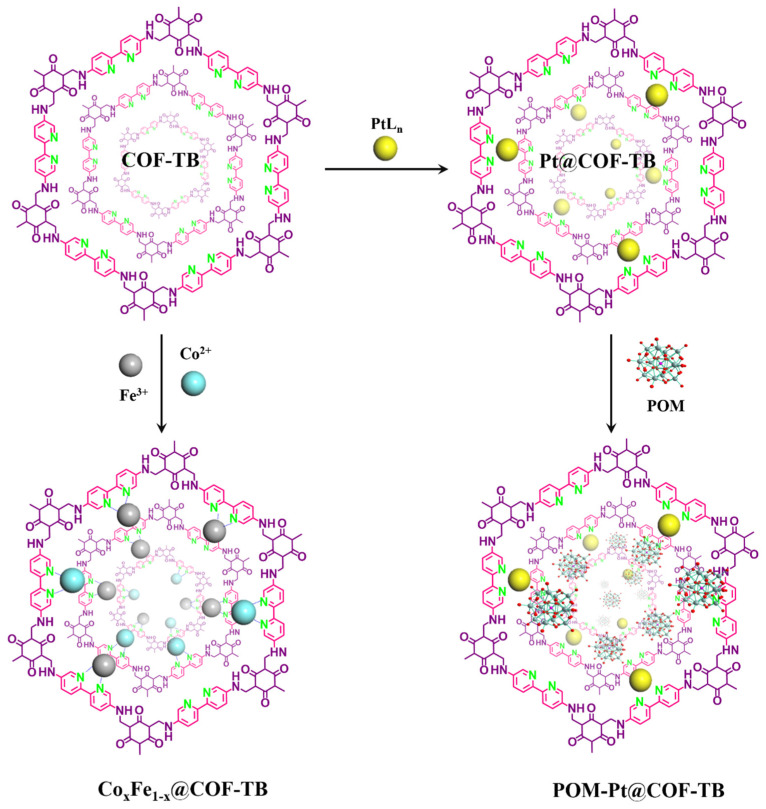
Synthesis route of metal COF composites.

The strategies for constructing metal composite catalysts Co_x_Fe_1–x_@COF–TB (x = 0, 0.25, 0.5, 0.75, 1.0) and POM–Pt@COF–TB using COF-TB as the matrix material are illustrated in Scheme 1. Because of the coordination interaction between nitrogen atoms and metal ions, COFs (COF–TB) with bipyridine groups within the backbone were selected for the construction of metal–loaded COF complexes. The COF–TB could be synthesized and prepared according to [37]. Then, the prepared COF–TB was processed with CoCl_2_ and FeCl_3_ in methanol for 12 h at room temperature to obtain a series of Co/Fe–based COFs composites in order to investigate the effect of different contents of Fe(III) and Co(II) polymetallic catalysts on the catalysis of OER. Furthermore, COF–TB was sequentially treated with Pt(DMSO)_2_Cl_2_ and POM in CH_2_Cl_2_ to construct the target material, POM–Pt@COF–TB. 11-Molybdo–1–vanadophosphoric acid was selected as the POM for this study. Various characterization methods were used to certify the successful synthesis of COF–TB and its metal compounds from different aspects.

**Figure 1:**
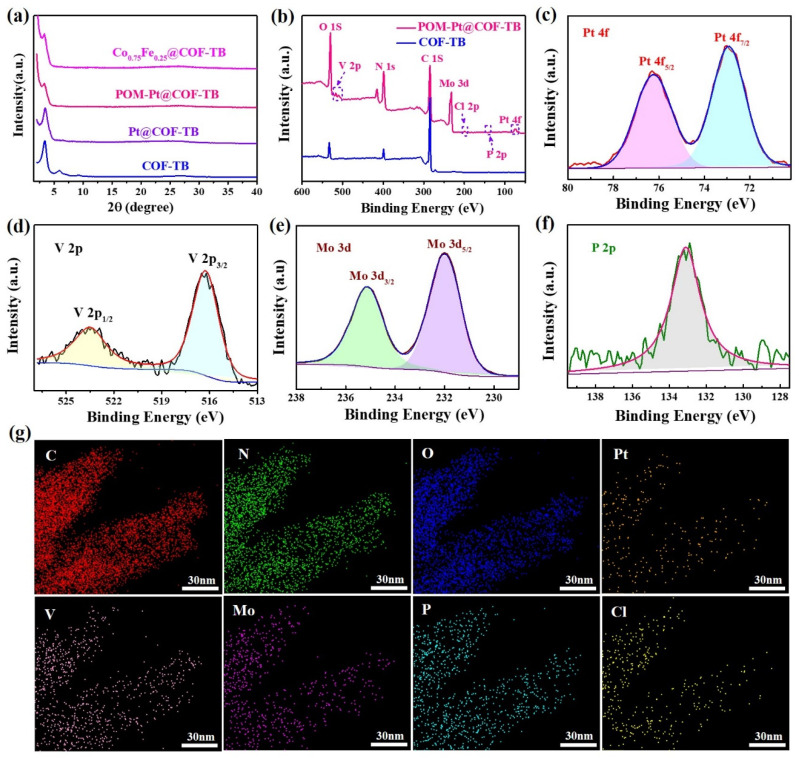
(**a**) Powder X–ray diffraction (PXRD) patterns of prepared COFs; XPS spectra of POM–Pt@COF–TB: (**b**) full elements; (**c**) Pt; (**d**) v; (**e**) Mo, (**f**) P; (**g**) EDS mapping images of POM–Pt@COF–TB.

In order to verify the crystal structure of the prepared COFs, powder X–ray diffraction (PXRD) analysis was carried out. Figure 1a exhibited that the PXRD diffraction of COF–TB was similar to that of a previously reported paper [38]. Meanwhile, the PXRD pattern of Pt@COF–TB and COF–TB displayed that the peak intensity of Pt@COF–TB decreased with the modification of Pt (Figure 1a). However, the main position at 3.46° (100) was still obvious, which verified that the crystal structure of Pt@COF–TB was still kept after the modification of Pt, allowing for the further introduction of POM. After further postmodification, the PXRD diffraction of POM–Pt@COF–TB displayed that the diffraction peaks of (100) still preserved, which further proved that the POM–modified COF composites still possessed a good crystal structure. Furthermore, the modification of POM and Pt could be verified by X–ray photoelectron spectroscopy (XPS) analysis. The result demonstrated that elements C, N, O, Pt, V, Cl, and Mo were clearly observed in the XPS spectra of POM–Pt@COF–TB (Figure 1b). The high–resolution Pt 4f spectrum showed two peaks with binding energies of 76.2 and 72.9 eV (Figure 1c) belonging to the 4f_7/2_ and 4f_2/5_ of Pt (II), respectively [10]. Figure 1d showed that there were two peaks at 523.5 and 516.3 eV that could be attributed to the binding energy of V 2p_1/2_ and V 2p_3/2_, indicating the presence of V^5+^ in the COF complexes [39]. In the high–resolution spectrum of Mo 3d (Figure 1e), the binding energies of Mo 3d_3/2_ and Mo 3d_5/2_ had two peaks at 235.2 and 232 eV. Meanwhile, the splitting width between them was 3.2 eV, which was consistent with the reported properties of Mo^6+^ [39]. A high–resolution P 2p spectrum appeared at 133.1 eV (Figure 1f), which is characteristic of P^5+^ in phosphates [40]. Furthermore, EDS mapping images of POM–Pt@COF–TB exhibited that the elements of C, N, O, Pt, V, Mo, P and Cl were uniformly distributed (Figure 1g and Figure S9). The Pt element was uniformly exposed on the surface of POM–Pt@COF–TB, which could offer more activity sites for the reaction.

Meanwhile, the Fourier transform infrared spectra (FT–IR) of the obtained materials were measured in order to verify the successful addition of metal and POM. The results in Figure S5 indicated that there were four characteristic peaks at 1062, 962, 866, and 781 cm^−1^ in the spectrum of POM, which were attributed to the stretching of P–O, Mo=O, interoctahedral Mo–O–Mo, and intraoctahedral Mo–O–Mo, respectively [41]. Compared to the FT–IR of COF–TB, the new stretching vibration peak of POM–Pt@COF–TB at 794 cm^−1^ may be caused by the stretching of Mo–O–Mo within the octahedra from the modified POM. Otherwise, compared with the FT–IR of the initial COF-TB, the FT–IR spectra of Pt@COF–TB and POM–Pt@COF–TB showed that the characteristic peaks present in the original COFs were conserved except for the red–shifted ν _C-N_ peak at 1259 cm^−1^, which further demonstrated the coordination of the metal with the bipyridyl nitrogen atoms in the COF backbone.

**Figure 2:**
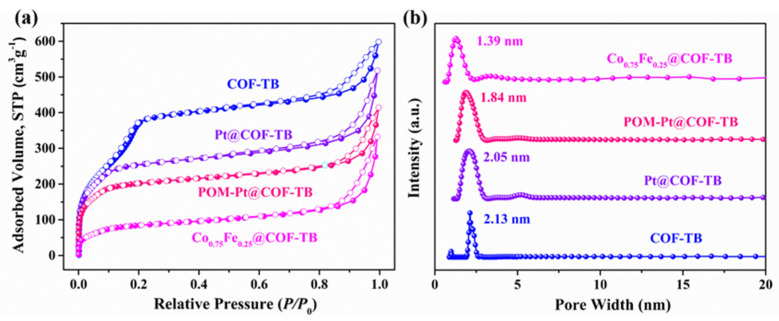
(**a**) Nitrogen adsorption–desorption isotherms of prepared COFs; (**b**) pore size distribution calculated with the NLDFT method.

Furthermore, the porosity data of prepared composites could be obtained from N_2_ adsorption measurements at 77 K according to the supplementary materials. On the one hand, the specific surface areas of COF–TB were reduced from 1216 to 965 m^2^ g^−1^, and the pore size distribution calculated by NLDFT method also decreased to 2.05 nm after the modification of Pt (Figure 2 and Figures S1–S3, Table 1). After the introduction of POM, the specific surface area of POM–Pt@COF–TB remained at 738 m^2^ g^−1^. The pore size distribution of POM–Pt@COF–TB was concentrated, and the pore size decreased to 1.84 nm compared to the initial COFs (Figure 2, Table 1). On the other hand, the specific surface areas of COF–TB decreased from 1216 to 319 m^2^ g^−1^ (Figure 2a and Figure S4) with the entry of Fe and Co into the pore structure of COFs. The pore size of COF–TB was decreased from 2.13 to 1.39 nm (Figure 2b and Table 1). The reduction in specific surface area and pore size may have been caused by the introduction of metals or POM in the pore channel. The single pore size distribution of the obtained COF–based composites demonstrates the uniformity of the modification.

To verify the catalytic performance of POM–Pt@COF–TB in the direct conversion of methane, the reaction was carried out at 60 °C according to the supplementary materials. H_2_O_2_ acted as the oxygen provider, and the POM might have acted as a cocatalyst in the reaction [42]. After that, the products of methane oxidation obtained at the same time interval were identified with ^1^H nuclear magnetic resonance spectroscopy (NMR). The results have been shown in Figure S10; the hydrogens of acetic acid (2.98 ppm) [43], methanol (3.34 ppm) [44], and ethanol (1.18 and 3.65 ppm) [45] were clearly visible after the catalysis of POM–Pt@COF–TB. Moreover, the content of methane oxidation products such as methanol and ethanol further increased with the progress of the catalytic reaction. After 6 h of reaction, the main catalytic product was acetic acid, which can probably be attributed to the further oxidation of ethanol. On the basis of these results, it can be inferred that POM–Pt@COF–TB achieved efficient catalytic performance in the direct methane conversion reaction, which may be attributed to the combined effect of the introduced Pt and POM.

**Figure 3:**
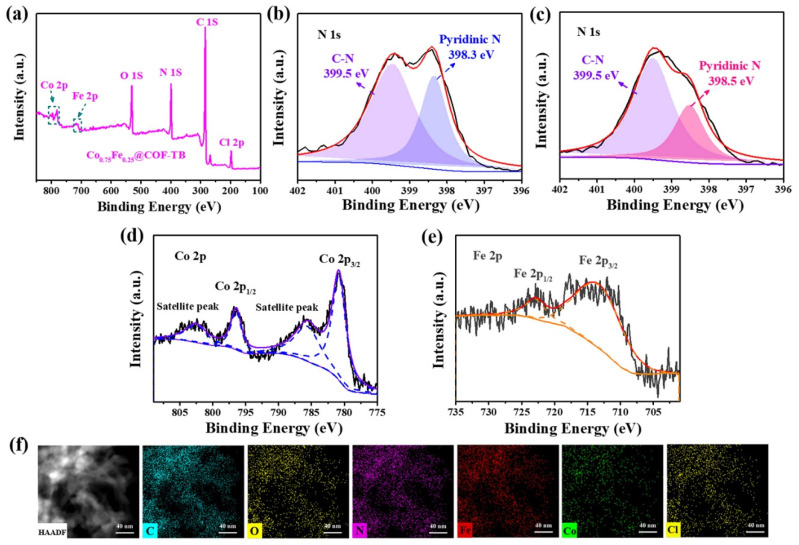
(**a**) XPS spectra of Co_0.75_Fe_0.25_@COF–TB; N 1s spectra of COF–TB (**b**) and Co_0.75_Fe_0.25_@COF–TB (**c**); Co 2p spectra (**d**) and Fe 2p spectra (**e**) of Co_0.75_Fe_0.25_@COF–TB; (**f**) EDS mapping images of Co_0.75_Fe_0.25_@COF–TB.

In addition, COF–TB was used as a matrix material to construct electrochemical OER catalysts and to obtain the bimetallic composite COF–TB composites. In the characterization of a Fe and Co bimetal-incorporated COF–TB composite, PXRD analysis was used to demonstrate the successful preparation of the composite for the first time. The result showed that the PXRD spectra of Co_0.75_Fe_0.25_@COF–TB that peaked at 3.46° belonging to the facet of (100) were still apparent compared with those of COF–TB, which suggested that the crystalline structure of the original COFs was still preserved after modification (Figure 1a). The FT–IR spectra of Co_0.75_Fe_0.25_@COF–TB also exhibited the same red–shift of ν _C-N_, which might be due to the coordination of the introduced Fe and Co ions with the bipyridine groups in the COFs (Figure S5). Meanwhile, the successful addition of the metal ions could be further certified by XPS analysis. Figure 1b displayed that the XPS spectra of COF–TB indicated the presence of only N, O, and C. Specifically, the N 1s spectra exhibited the presence of two distinct peaks at 399.5 and 398.3 eV associated with C–N and pyridinic nitrogen, respectively (Figure 3b). After the impregnation of iron and cobalt, the shake–up satellite signal in Co 2p and Fe 2p spectra clearly illustrated the existence of Co and Fe in Co_0.75_Fe_0.25_@COF–TB (Figure 3a). For the high–resolution Co 2p spectra, the major peaks at about 796.4 and 780.9 eV were assigned to Co 2p_1/2_ and Co 2p_3/2_, respectively (Figure 3d) [46,47]. The Co^2+^ oxidation state was confirmed in the Co_0.75_Fe_0.25_@COF–TB. Furthermore, the Fe^3+^ oxidation state could be verified with the signals of Fe 2p_1/2_ and Fe 2p_3/2_ located at 780.9 and 796.4 eV, respectively (Figure 3e) [12]. During the impregnation of cobalt and iron, a shift in the binding energy of pyridinic nitrogen (from 398.3 to 398.5 eV) was also observed (Figure 3b,c). However, the peak position of C–N (399.5 eV) did not change, which certified that the complexation of COF–TB with cobalt and iron was only carried out through its bipyridine units.

Moreover, the pore structure can be viewed clearly in the transmission electron microscopy (TEM) images of Co_0.75_Fe_0.25_@COF–TB (Figure S6). The one–dimensional channels with regularity can provide a pathway for the free flow of water molecules. Figure 3f showed that the distribution of C, O, N, Fe, and Co was uniform on the whole, which indicated that the synthesized COF composites were homogeneous. The Co and Fe elements did not aggregate and were completely exposed on the surface of Co_0.75_Fe_0.25_@COF–TB, which provided more active sites for the electrocatalytic OER. Moreover, the EDS spectra of Co_0.75_Fe_0.25_@COF–TB displayed that the loading of Co and Fe accounted for 12.65 and 3.09 wt % of the total COF, respectively (Figure S7). These results indicated the successful modification of metals in COFs, and the electrocatalytic properties of composites were then measured.

**Figure 4:**
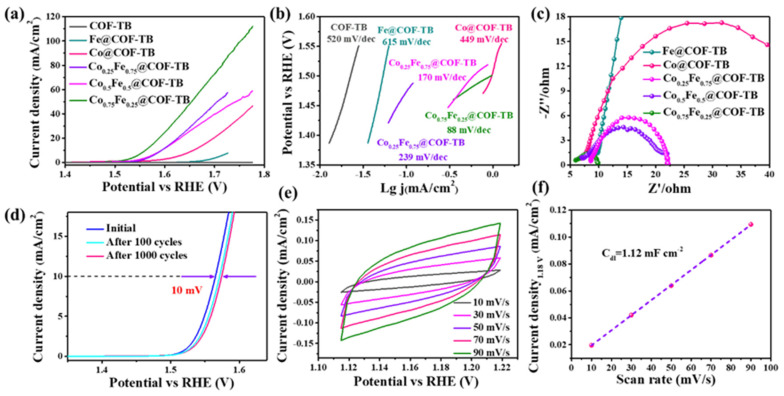
(**a**) LSV curves toward OER of COF–TB, Co_x_Fe_1–x_@COF–TB (x = 0, 0.25, 0.5, 0.75, 1); (**b**) Tafel slopes of the prepared materials; (**c**) Nyquist plots of Co_x_Fe_1–x_@COF–TB (x = 0, 0.25, 0.5, 0.75, 1); (**d**) LSV curves for Co_0.75_Fe_0.25_@COF–TB before and after 100 and 1000 cycles of CV scans; (**e**) CV plots of Co_0.75_Fe_0.25_@COF–TB tested at various scan rates from 10 to 90 mV s^−1^; (**f**) ECSA evaluation.

To explore the electrocatalytic performance of the Co/Fe–based COF composites, the oxygen precipitation reaction of the prepared metal–loaded COF composites was measured using a 1.0 M KOH solution as the electrolyte. First, the LSV curve of COF–TB was almost unchanged when the voltage rose to 1.7 vs. the reversible hydrogen electrode (RHE) (Figure 4a). The comparison of the LSV curves of other materials clear exhibited that the primitive COF–TB did not contribute to the electrocatalytic OER. After the coordination of metal ions, the current density was increased in different degrees. In the case of single–metal modification, the OER activity of the COF composites with the addition of Co was higher than that of Fe. Meanwhile, considering that the addition of an appropriate quantity of Fe can motivate strong electronic interactions between Fe and Co, the OER of bimetallic COFs with different counts of Fe was also measured. Figure 4a displayed that the overpotentials of Co_0.25_Fe_0.75_@COF–TB (364 mV), Co_0.5_Fe_0.5_@COF–TB (362 mV), and Co_0.75_Fe_0.25_@COF-TB (331 mV) were much lower than those of Co@COF–TB (427.8 mV) and Fe@COF–TB, which might be due to the significant synergistic effect between Co and Fe. Additionally, the Tafel slope of Co_0.75_Fe_0.25_@COF–TB was 88 mV dec^−1^, which was also much smaller than that of the others, and exhibited faster reaction kinetics and better electrocatalytic properties (Figure 4b).

Furthermore, the charge transfer ability of the surface of COFs also impacted their catalytic performance. Therefore, the electrochemical impedance spectroscopy (EIS) of metal coordinated COF composites were also tested. Figure S11 showed that the resistance of the charge transfer of Co@COF–TB was much smaller than that of the original COF–TB and Fe@COF–TB. Figure 4c displayed that the charge transfer resistance of Co@COF–TB could be effectively reduced with the further introduction of Fe. Among the metal COF composites with different amounts of bimetallic modification, the charge transfer resistance of Co_0.75_Fe_0.25_@COF–TB was the smallest (Figure 4c and Figure S11), which further explicated the better OER activity of Co_0.75_Fe_0.25_@COF–TB. The double–layer capacitor (C_dl_) of Co_0.75_Fe_0.25_@COF–TB was 1.12 mF cm^−2^ (Figure 4f), and the electrochemically active surface area (ECSA), calculated according to Figure 4e, was 28 cm^−2^ ECSA. The larger value of ECSA allowed for the active sites on the surface to be more efficiently in contact with water molecules for electron transfer. The electrochemical stability of Co_0.75_Fe_0.25_@COF–TB was also measured under the same conditions, and the results show that the overpotential of Co_0.75_Fe_0.25_@COF–TB increased by 7 mV after 100 cycles, and only 3 mV after another 1000 cycles (Figure 4d), which demonstrated that the composite catalyst exhibited great stability in the oxygen reduction process. Meanwhile, with cobalt and iron ions as the active sites, the turnover frequency (TOF) of Co_0.75_Fe_0.25_@COF–TB at an overpotential of 330 mV was 0.119 s^−1^, which indicated highly efficient metal atom utilization and better than that of other metal–containing COFs catalysts such as COF–TpDb–TZ–Co [23], Co@COF–Pyr [48], Co_0.5_V_0.5_@COF–SO_3_ [36] and Co–TpBpy [38] (Table S1). Furthermore, the relationship between TOF value and overpotential is visualized in Figure S12, showing a positive correlation between TOF and current density. On the whole, Co_0.75_Fe_0.25_@COF–TB with a larger specific surface area exhibited better electron transfer capability and OER performance.

## 3. Materials and Methods

### 3.1. Materials and Reagents

2,4,6–Trihydroxybenzene–1,3,5–tricarbaldehyde was ordered from Jilin Yanshen Technology Co., Ltd., Jilin, China. 2,2’–Bipyridine–5,5’–diamine, acetic acid (99%) was obtained from Bide Pharmatech Co., Ltd., Shanghai, China. 1,3,5-Trimethylbenzene (97%), 1,4–dioxane (97%), potassium tetrachloroplatinate(II) (K2PtCl4, 98%), deuterium oxide (99.9%), sodium metavanadate (99.0%), sodium phosphate dibasic dodecahydrate (99%), and sodium molybdate dihydrate (99.0%) were prepared from Aladdin Co., Ltd., Shanghai, China. A total of 30 wt % H_2_O_2_ aqueous water, dimethylformamide (DMF, ≥99.5%), isopropyl alcohol (≥99.7%), and methanol (≥99.5%) were bought from Xilong Scientific Co., Ltd., Shantou, China. Iron(III) chloride hexahydrate and cobalt(II) chloride were obtained from Shanghai Macklin Biochemical Co., Ltd., Shanghai, China. The preparation of COF–TB [37] and 11–molybdo–1–vanadophosphoric acid (polyoxometalates, POM) [49,50] was based on the supplementary materials. The preparation of COF–TB needs to be carried out at 120 °C without water and oxygen, while the preparation of POM needs to be carried out under normal conditions. The prepared products were dried under vacuum overnight without special instructions.

### 3.2. Synthesis of Co_x_Fe_1-x_@COF–TB (x = 0, 0.25, 0.5, 0.75, 1)

COF–TB (10 mg) was impregnated in a mixture of 15.3x mg CoCl_2_ and 9.95 (1 – x) mg FeCl_3_ prepared with anhydrous methanol as the solvent. Then, the solution was stirred overnight and washed with methanol. Co_x_Fe_1–x_@COF–TB (x = 0, 0.25, 0.5, 0.75, 1) could be obtained after being dried for 6 h.

### 3.3. Synthesis of Pt@COF–TB and POM–Pt@COF–TB

Pt(DMSO)_2_Cl_2_ (4.68 mg, 0.01 mmol) and COF–TB were dispersed in 4 mL of anhydrous dichloromethane, and the mixture was stirred at room temperature for 24 h. After being centrifuged, the collected solids were washed and extracted in a Soxhlet extractor with dichloromethane. Lastly, the powder Pt@COF–TB was collected after having been dried overnight at 60 °C.

For the preparation of POM–Pt@COF–TB, POM (60.83 mg) was dissolved in ultrapure water (20 mL). Then, Pt@COF–TB was impregnated in the mixture solution and stirred at room temperature for 12 h. After being washed with ultrapure water, the powder (POM–Pt@COF–TB) was obtained after having been dried.

## 4. Conclusions

In conclusion, a series of novel COF composites containing a bipyridine multimetal complex were designed and synthesized. Specifically, the Pt and POM–modified COFs (POM–Pt@COF–TB) exhibited great catalysis for methane conversion. In addition, a series of Co/Fe-based COFs materials were fabricated. The optimized bimetallic composite (Co_0.75_Fe_0.25_@COF–TB) possessed low overpotential and excellent stability. The overpotential of Co_0.75_Fe_0.25_@COF–TB was 331 mV in a 1 M KOH solution at a current density of 10 mA cm^−2^. Remarkably, its great performance remained after 1000 CV scans, which might have been due to the better utilization of the loaded cobalt by the introduction of iron. Co_0.75_Fe_0.25_@COF–TB exhibited a great TOF value (0.119 s^−1^) at the overpotential of 330 mV, which exhibited high efficiency in the utilization of metal atoms and was better than that of many reported COFs–based OER electrocatalysts. This work provides a new perspective for the future coordination of COFs with bimetallic or polymetallic ions, and broadens the application of COFs in methane conversion and electrocatalytic oxygen evolution.

## Figures and Tables

**Table 1:** Porosity data of prepared porous materials.

COFs	S_BET_ (m^2^ g^−1^) ^a^	S_L_ (m^2^ g^−1^) ^b^	V_total_ (cm^3^ g^−1^) ^c^	D_pore_ (nm) ^d^
COF–TB	1216	1263	1.002	2.13
Pt@COF–TB	965	1207	0.8008	2.05
POM–Pt@COF–TB	738	892	0.6408	1.84
Co_0.75_Fe_0.25_@COF–TB	319	369	0.5138	1.39

^a^ Specific surface area calculated by the BET method; ^b^ specific surface area calculated via the Langmuir method; ^c^ total pore volume at *P/P*_0_ = 0.99; ^d^ data calculated with the NLDFT method.

## Data Availability

Not applicable.

## Supplementary Materials

The following supporting information can be downloaded at: https://www.mdpi.com/article/10.3390/molecules27165193/s1. Scheme S1: synthesis of COF–TB; Figure S1: BET specific surface area plot of COF–TB; Figure S2: BET specific surface area plot of Pt@COF–TB; Figure S3: BET specific surface area plot of POM–Pt@COF–TB; Figure S4: BET specific surface area plot of Co_0.75_Fe_0.25_@COF–TB; Figure S5: FT-IR spectra of POM, COF–TB, Pt@COF-TB, POM-Pt@COF–TB and Co_0.75_Fe_0.25_@COF–TB; Figure S6: TEM of Co_0.75_Fe_0.25_@COF–TB; Figure S7: EDS spectra of Co_0.75_Fe_0.25_@COF-TB; Figure S8: TEM of POM–Pt@COF–TB; Figure S9: EDS spectra of POM–Pt@COF–TB; Figure S10: ^1^H NMR spectra of the methane oxidation reactions products; Figure S11: Nyquist plots of COF–TB, Fe@COF–TB and Co@COF–TB; Figure S12: TOF value vs. overpotential of Co_0.75_Fe_0.25_@COF–TB; Table S1: comparison of the reported OER catalytic performance of metal–containing COFs catalysts.

## Sample Availability

Samples of the compounds are available from the authors.

## References

[1] Losantos R., Sampedro D. (2021). Design and tuning of photoswitches for solar energy storage. Molecules.

[2] Lazzarin L., Pasini M., Menna E. (2021). Organic functionalized carbon nanostructures for solar energy conversion. Molecules.

[3] Tang S., Zeng L., Lei A. (2018). Oxidative R^1^–H/R^2^–H cross-coupling with hydrogen evolution. J. Am. Chem. Soc..

[4] Pradeep N., Tamil Selvi G., Venkatraman U., Van Le Q., Jeong S.K., Pandiaraj S., Alodhayb A., Muthuramamoorthy M., Grace A.N. (2021). Development and investigation of the flexible hydrogen sensor based on ZnO-decorated Sb_2_O_3_ nanobelts. Mater. Today Chem..

[5] Sorcar S., Das J., Komarala E.P., Fadeev L., Rosen B.A., Gozin M. (2022). Design of coke-free methane dry reforming catalysts by molecular tuning of nitrogen-rich combustion precursors. Mater. Today Chem..

[6] Connolly B.M., Aragones-Anglada M., Gandara-Loe J., Danaf N.A., Lamb D.C., Mehta J.P., Vulpe D., Wuttke S., Silvestre-Albero J., Moghadam P.Z. (2019). Tuning porosity in macroscopic monolithic metal-organic frameworks for exceptional natural gas storage. Nat. Commun..

[7] Yu J., He Q., Yang G., Zhou W., Shao Z., Ni M. (2019). Recent advances and prospective in ruthenium-based materials for electrochemical water splitting. ACS Catal..

[8] Bai L., Duan Z., Wen X., Si R., Zhang Q., Guan J. (2019). Highly dispersed ruthenium-based multifunctional electrocatalyst. ACS Catal..

[9] Jensen A.W., Sievers G.W., Jensen K.D., Quinson J., Arminio-Ravelo J.A., Brüser V., Arenz M., Escudero-Escribano M. (2020). Self-supported nanostructured iridium-based networks as highly active electrocatalysts for oxygen evolution in acidic media. J. Mater. Chem. A.

[10] Xie P., Pu T., Nie A., Hwang S., Purdy S.C., Yu W., Su D., Miller J.T., Wang C. (2018). Nanoceria-supported single-atom platinum catalysts for direct methane conversion. ACS Catal..

[11] Tomkins P., Ranocchiari M., van Bokhoven J.A. (2017). Direct conversion of methane to methanol under mild conditions over cu-zeolites and beyond. Acc. Chem. Res..

[12] Feng X., Gao Z., Xiao L., Lai Z., Luo F. (2020). A Ni/Fe complex incorporated into a covalent organic framework as a single-site heterogeneous catalyst for efficient oxygen evolution reaction. Inorg. Chem. Front..

[13] Fernandes S.P.S., Mellah A., Kovář P., Sárria M.P., Pšenička M., Djamila H., Salonen L.M., Espiña B. (2020). Extraction of ibuprofen from natural waters using a covalent organic framework. Molecules.

[14] Liu X., Xiao S., Jin T., Gao F., Wang M., Gao Y., Zhang W., Ouyang Y., Ye G. (2022). Selective entrapment of thorium using a three-dimensional covalent organic framework and its interaction mechanism study. Sep. Purif. Technol..

[15] Zhao Y., Sui Z., Chang Z., Wang S., Liang Y., Liu X., Feng L., Chen Q., Wang N. (2020). A trifluoromethyl-grafted ultra-stable fluorescent covalent organic framework for adsorption and detection of pesticides. J. Mater. Chem. A.

[16] Liu X., Wang S., Liang Y., Zhao Y., Yuan N., Sui Z., Chen Q. (2021). Adenine-bearing covalent organic frameworks via one-pot tandem reaction for selective adsorption of Ag+. Microporous Mesoporous Mater..

[17] Zhang S., Liu D., Wang G. (2022). Covalent organic frameworks for chemical and biological sensing. Molecules.

[18] Lu Y., Liang Y., Zhao Y., Xia M., Liu X., Shen T., Feng L., Yuan N., Chen Q. (2021). Fluorescent test paper via the in situ growth of COFs for rapid and convenient detection of Pd(II) ions. ACS Appl. Mater. Interfaces.

[19] Skorjanc T., Shetty D., Valant M. (2021). Covalent organic polymers and frameworks for fluorescence-based sensors. ACS Sens..

[20] Baldwin L.A., Crowe J.W., Shannon M.D., Jaroniec C.P., McGrier P.L. (2015). 2D covalent organic frameworks with alternating triangular and hexagonal pores. Chem. Mater..

[21] Liang Y., Xia M., Zhao Y., Wang D., Li Y., Sui Z., Xiao J., Chen Q. (2022). Functionalized triazine-based covalent organic frameworks containing quinoline via aza-Diels-Alder reaction for enhanced lithium-sulfur batteries performance. J. Colloid Interface Sci..

[22] Liu X., Xia M., Zhao Y., Xia T., Li Y., Xiao J., Sui Z., Chen Q. (2022). Cationic covalent organic framework via cycloaddition reactions as sulfur-loaded matrix for lithium-sulfur batteries. Mater. Today Chem..

[23] Liang Y., Xia T., Wu Z., Yang Y., Li Y., Sui Z., Li C., Fan R., Tian X., Chen Q. (2022). Tetrazole-functionalized two-dimensional covalent organic frameworks coordinated with metal ions for electrocatalytic oxygen evolution reaction. Mater. Today Chem..

[24] Xie M., Li C., Ren S., Ma Y., Chen X., Fan X., Han Y., Shi Z., Feng S. (2022). Ultrafine Sb nanoparticles in situ confined in covalent organic frameworks for high-performance sodium-ion battery anodes. J. Mater. Chem. A.

[25] Calik M., Auras F., Salonen L.M., Bader K., Grill I., Handloser M., Medina D.D., Dogru M., Löbermann F., Trauner D. (2014). Extraction of photogenerated electrons and holes from a covalent organic framework integrated heterojunction. J. Am. Chem. Soc..

[26] Chen L., Furukawa K., Gao J., Nagai A., Nakamura T., Dong Y., Jiang D. (2014). Photoelectric covalent organic frameworks: Converting open lattices into ordered donor–acceptor heterojunctions. J. Am. Chem. Soc..

[27] Ren X., Liao G., Li Z., Qiao H., Zhang Y., Yu X., Wang B., Tan H., Shi L., Qi X. (2021). Two-dimensional MOF and COF nanosheets for next-generation optoelectronic applications. Coord. Chem. Rev..

[28] Keller N., Bein T. (2021). Optoelectronic processes in covalent organic frameworks. Chem. Soc. Rev..

[29] Li J., Zhao D., Liu J., Liu A., Ma D. (2020). Covalent organic frameworks: A promising materials platform for photocatalytic CO_2_ reductions. Molecules.

[30] Li Y., Zuo K., Gao T., Wu J., Su X., Zeng C., Xu H., Hu H., Zhang X., Gao Y. (2022). Bimetallic docked covalent organic frameworks with high catalytic performance towards coupling/oxidation cascade reactions. RSC Adv..

[31] Chai Y., Li Y., Hu H., Zeng C., Wang S., Xu H., Gao Y. (2021). N-heterocyclic carbene functionalized covalent organic framework for transesterification of glycerol with dialkyl carbonates. Catalysts.

[32] Zhang B., Wang W., Liang L., Xu Z., Li X., Qiao S. (2021). Prevailing conjugated porous polymers for electrochemical energy storage and conversion: Lithium-ion batteries, supercapacitors and water-splitting. Coord. Chem. Rev..

[33] Vardhan H., Pan Y., Yang Z., Verma G., Nafady A., Al-Enizi A.M., Alotaibi T.M., Almaghrabi O.A., Ma S. (2019). Iridium complex immobilization on covalent organic framework for effective C—H borylation. APL Mater..

[34] Liang Z., Wang H.-Y., Zheng H., Zhang W., Cao R. (2021). Porphyrin-based frameworks for oxygen electrocatalysis and catalytic reduction of carbon dioxide. Chem. Soc. Rev..

[35] Guan Y., Lai J., Xu G. (2021). Recent advances on electrocatalysis using pristinely conductive metal-organic frameworks and covalent organic frameworks. ChemElectroChem.

[36] Gao Z., Yu Z., Huang Y., He X., Su X., Xiao L., Yu Y., Huang X., Luo F. (2020). Flexible and robust bimetallic covalent organic frameworks for the reversible switching of electrocatalytic oxygen evolution activity. J. Mater. Chem. A.

[37] Sun Q., Aguila B., Perman J., Nguyen N., Ma S. (2016). Flexibility matters: Cooperative active sites in covalent organic framework and threaded ionic polymer. J. Am. Chem. Soc..

[38] Aiyappa H.B., Thote J., Shinde D.B., Banerjee R., Kurungot S. (2016). Cobalt-modified covalent organic framework as a robust water oxidation electrocatalyst. Chem. Mater..

[39] Barats-Damatov D., Shimon L.J.W., Feldman Y., Bendikov T., Neumann R. (2015). Solid-state crystal-to-crystal phase transitions and reversible structure–temperature behavior of phosphovanadomolybdic acid, H_5_PV_2_Mo_10_O_40_. Inorg. Chem..

[40] Predoeva A., Damyanova S., Gaigneaux E.M., Petrov L. (2007). The surface and catalytic properties of titania-supported mixed PMoV heteropoly compounds for total oxidation of chlorobenzene. Appl. Catal. A.

[41] Chavan L.D., Shankarwar S.G. (2015). KSF supported 10-molybdo-2-vanadophosphoric acid as an efficient and reusable catalyst for one-pot synthesis of 2,4,5-trisubstituted imidazole derivatives under solvent-free condition. Chin. J. Catal..

[42] Bar-Nahum I., Khenkin A.M., Neumann R. (2004). Mild, aqueous, aerobic, catalytic oxidation of methane to methanol and acetaldehyde catalyzed by a supported bipyrimidinylplatinum-polyoxometalate hybrid compound. J. Am. Chem. Soc..

[43] Shavi R., Ko J., Cho A., Han J.W., Seo J.G. (2018). Mechanistic insight into the quantitative synthesis of acetic acid by direct conversion of CH_4_ and CO_2_: An experimental and theoretical approach. Appl. Catal. B.

[44] Saveant J.-M., Tard C. (2016). Attempts to catalyze the electrochemical CO_2_-to-methanol conversion by biomimetic 2e^−^ + 2H^+^ transferring molecules. J. Am. Chem. Soc..

[45] Ghanghas R., Jindal A., Vasudevan S. (2020). Geometry of hydrogen bonds in liquid ethanol probed by proton NMR experiments. J. Phys. Chem. B.

[46] Chandra S., Kundu T., Dey K., Addicoat M., Heine T., Banerjee R. (2016). Interplaying intrinsic and extrinsic proton conductivities in covalent organic frameworks. Chem. Mater..

[47] Elko-Hansen T.D.M., Ekerdt J.G. (2014). XPS investigation of the atomic layer deposition half reactions of bis(N-tert-butyl-N′-ethylpropionamidinato) cobalt(II). Chem. Mater..

[48] Zhao Y., Yang Y., Xia T., Tian H., Li Y., Sui Z., Yuan N., Tian X., Chen Q. (2021). Pyrimidine-functionalized covalent organic framework and its cobalt complex as an efficient electrocatalyst for oxygen evolution reaction. ChemSusChem.

[49] Tsigdinos G.A., Hallada C.J. (1968). Molybdovanadophosphoric acids and their salts. I. Investigation of methods of preparation and characterization. Inorg. Chem..

[50] Xia M., Qiu L., Li Y., Shen T., Sui Z., Feng L., Chen Q. (2022). A metal-organic frameworks composite catalyst containing platinum and polyoxometalate for direct conversion of methane. Mater. Lett..

