# Supplementary material for: Covalent Organic Frameworks Composites Containing Bipyridine Metal Complex for Oxygen Evolution and Methane Conversion
Source: Molecules. 2022 Aug 15;27(16):5193. doi: 10.3390/molecules27165193 (PMC9416349; doi:10.3390/molecules27165193)
Supplement: Supplementary file 1 [file molecules-27-05193-s001.zip › molecules-1836444-supplementary.pdf]

Supplementary materials for

Covalent Organic Frameworks Composites Containing Bipyridine Metal Complex for Oxygen Evolution and Methane Conversion

Xin Liu 1,2, Lijuan Feng 1,*, Yongpeng Li 3, Tian Xia 2, Zhuyin Sui 3,* and Qi Chen 2,*

1 Department of Bioengineering, Zhuhai Campus of Zunyi Medical University, Zhuhai 519041, China

2 State Key Laboratory of Marine Resource Utilization in South China Sea, Hainan University, Haikou 570228, China

3 School of Chemistry & Chemical Engineering, Yantai University, Yantai 264005, China

*** Correspondence: fljcyczh@126.com (L.F.); suizy@ytu.edu.cn (Z.S.); chenqi@hainanu.edu.cn (Q.C.)**

S1. Characterization methods and instruments

If not specifically described, all experiments were performed under nitrogen atmosphere. The Powder X-ray diffraction (PXRD) of COF-TB, Pt@COF-TB and $Co_{0.75}Fe_{0.25}$@COF-TB were measured on Bruker AXS D8 Advance Labx diffractometer at 40 kV and 30 mA using Cu Kα radiation over a 2θ range of 2-40°. And the X-ray photoelectron spectroscopy (XPS) measurement was carried out on an EscaLab 250Xi spectrometer (Thermo Fisher Scientific, USA). The field-emission transmission electron (FE-TEM) images and the corresponding EDS elemental mapping images (data) of all the obtained COFs were measured on a FEI Tecnai G2 F20 spectrometer. After being degassed at 110°C for 10 hours, the nitrogen adsorption isotherms of COF-TB, Pt@COF-TB, POM-Pt@COF-TB and $Co_{0.75}Fe_{0.25}$@COF-TB were performed with Micromeritics ASAP 2460 automated sorption analyzer. And the BET specific surface area of the synthesized COFs were calculated on the basis of the obtained adsorption−desorption isotherms. The pore size distribution profiles were calculated from the related adsorption branches by the nonlocal density function theory (NLDFT) approach. The total pore volume was calculated by the nitrogen adsorption-desorption isotherm at $P/P_0 = 0.99$.

S2. Synthetic Procedures

Synthesis of COF-TB

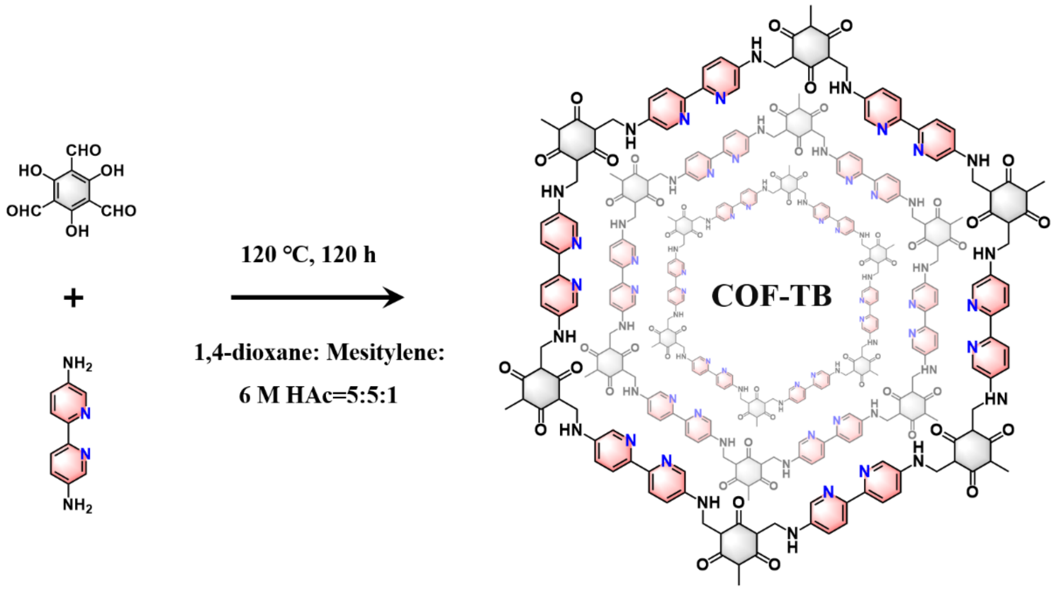

Scheme S1. Synthesis of **COF-TB**.

This compound was prepared by the followed pocedures according to the described in the literature. (Scheme S1.) 2,4,6-trihydroxybenzene-1,3,5-tricarbaldehyde (27.9 mg, 0.15 mmol), 2,2'-Bipyridine-5,5'-diamine (21 mg, 0.01 mmol) were added to a pyrex tube containing a mixture of 1,3,5-trimethylbenzene (0.5 ml) and 1,4-dioxane (0.5 ml). After being ultrasonically dispersed for 10 minutes, 6M acetic-acid catalyst (0.1ml) were added to the pyrex tube. Then, the pyrex tube was degassed via three freeze–pump–thaw cycles and sealed off by flame. After being heated at 120°C for five days, the product was washed with THF and then subjected to Soxhlet extraction with THF as the solvent. The powder was dried under vacuum and obtain the COF-TB (42.4 mg).

Synthesis of ***11-Molybdo-1-vanadophosphoric Acid (Polyoxometalates, POM)***

Disodium hydrogen phosphate dodecahydrate (5.97 g) was dissolved in 34 mL of water and mixed with sodium metavanadate (8.12 g) that had been dissolved by boiling in 34 mL of water. After cooling to ambient temperature, 1.6 mL concentrated sulfuric acid was added. To this mixture was added an aqueous solution of sodium molybdate (40.33 g), and while 28 mL of concentrated sulfuric acid was added slowly with vigorous stirring of the solution. The formed products were extracted with ether (166.7

mL). After separation, the ether was removed, and the remaining solid was dissolved in water, recrystallized, washed, and air-dried to obtain polyoxometalates.

S3. Study on the OER performances of the prepared catalysts

The OER performances of the prepared polymetallic modified COFs composites were measured by the Gamry 1010E electrochemical workstation with a PINE rotating disk electrode (RDE 710). And the 5 mm glassy carbon electrode (GCE), Hg/HgO electrode and graphite rod electrode were used as working electrode, reference electrode and counter electrode, respectively. And The GCE was polished with 1 μm, 0.3 μm, 0.05 μm alumina powder before the electrochemical measurements, respectively. And the catalyst slurry was prepared by dissolving catalyst (4.0 mg) and carbon black (1 mg) into 1 mL solution including 0.95 mL ethanol and 50 μL Nafion solution, and then the mixture was ultrasonically dispersed for 30 minutes. Finally, the prepared slurry was dropped onto the GCE to obtain the film electrode. For the OER experiment, CV scans of the GCE electrodes covered with the catalysts were carried out at a scan rate of 50 $mV \cdot s^{-1}$ until the current was stable. Then, LSV test was carried out at the scanning rate of 5 $mV \cdot s^{-1}$ in 1 M KOH electrolyte to determine the performance of OER. Moreover, the electrochemically active surface area (ECSA) of $Co_{0.75}Fe_{0.25}$@COF-TB was determined based on the double-layer capacitance (C_{dl}) using a simple cyclic voltammetry method in a non-Faradic potential range of 1.10-1.29 V vs RHE. The linear trend of capacitance density with scanning rate can be found by plotting the relationship between capacitance densities and scanning rate (10, 30, 50, 70 and 90 mV s^{-1}) at 1.18 V vs RHE. Durability test was carried out mainly through accelerated durability test (ADT) at 100 mV s^{-1}, and the potential was cycled from 1.43 V/RHE to 1.58 V/RHE for 1000 cycles. The slope of the fitted line is the twice of C_{dl}.

S4. Study on the catalytic conversion of methane

The CH_4 oxidation reaction was implemented in a stainless-steel autoclave containing a Teflon liner container (working volume, 50 mL). Specifically, 5 mL hydrogen peroxide (30%) and 10 mg catalyst were mixed in the container firstly. Then the autoclave was flushed with CH_4 three times and pressurized to 50 bar methane. Finally, the reaction mixture was heated to the required temperature (60 °C). The liquid

products of methane oxidation were identified by ^{1}H NMR on Bruker AVANCE NEO400 using deuterium oxide as the solvent. It should be noted that we dissolved the same amount of product in a certain amount of deuterium oxide solvent during the preparation of the sample before testing, and then the treated sample was subsequently tested by ^{1}H NMR on Bruker AVANCE NEO400.

S5. Calculation formula for the turnover frequency (TOF) of $Co_{0.75}Fe_{0.25}$@COF-TB

The TOF of $Co_{0.75}Fe_{0.25}$@COF-TB was calculated by the following equation:

$$\mathrm{TOF} = \frac{JA}{4Fn}$$

In the formula, J (A cm^{-2}) is the current density at overpotential of 331 mV, F is Faraday constant and n stands for the number of electrons transferred during OER.

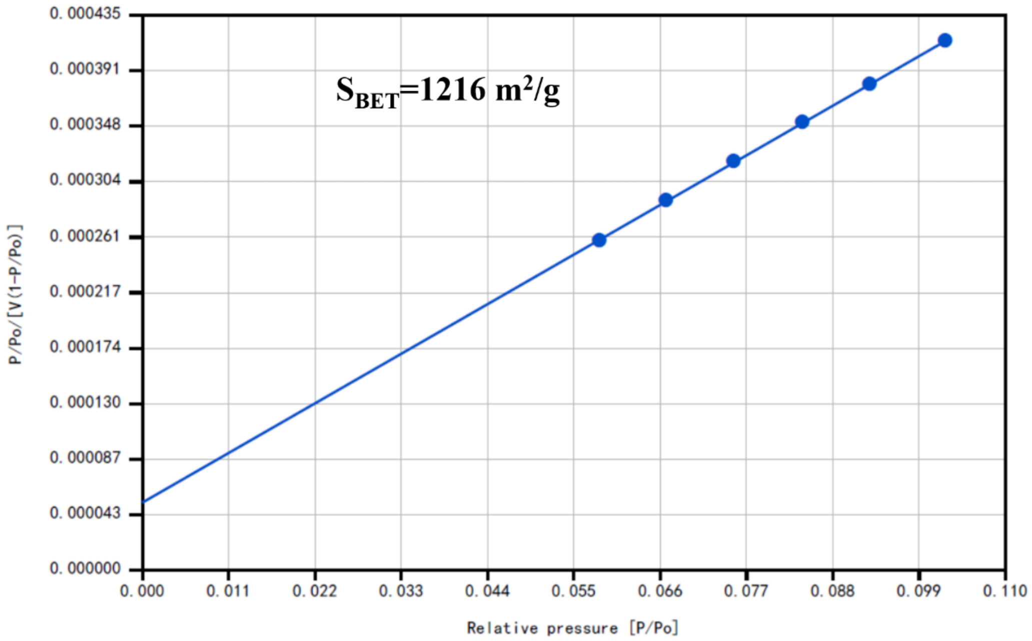

Figure S1. BET specific surface area plot of COF-TB.

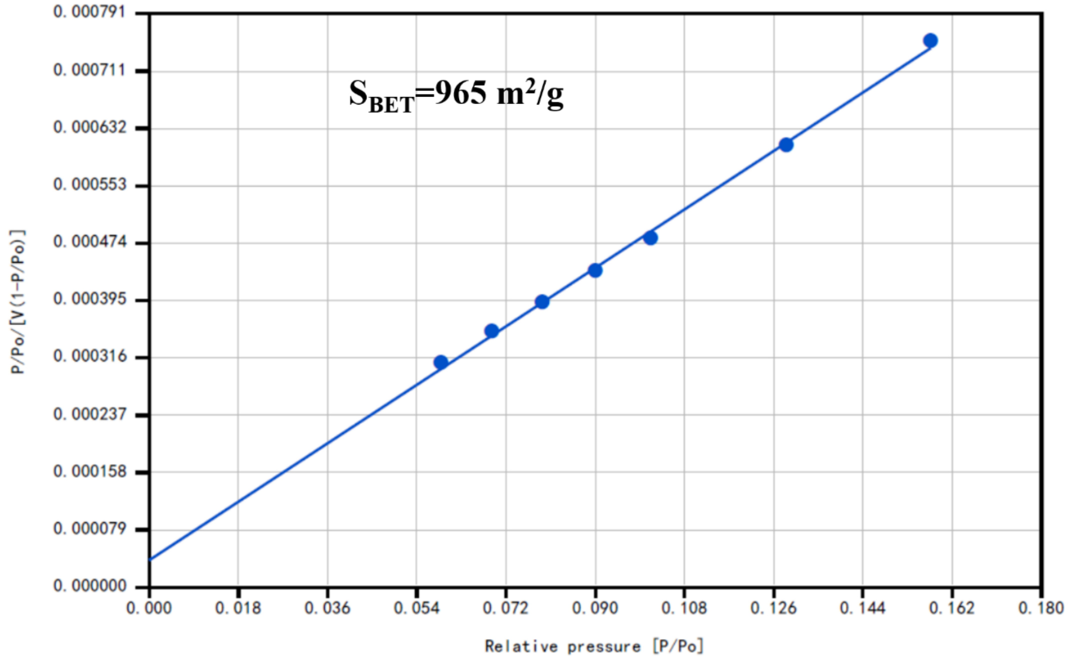

Figure S2. BET specific surface area plot of Pt@COF-TB.

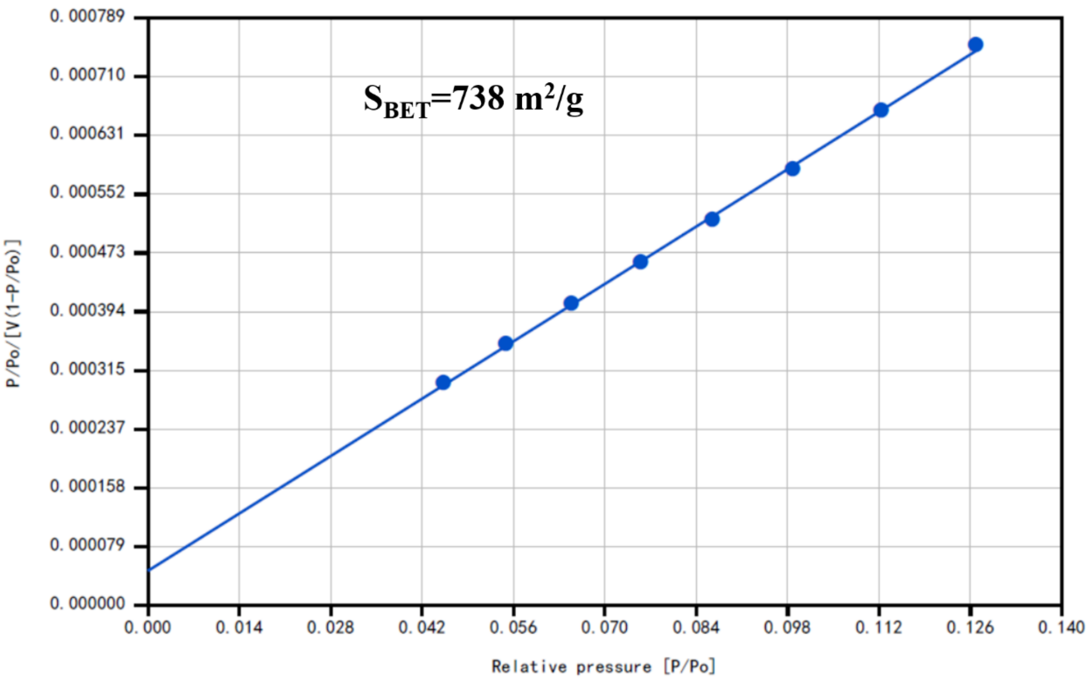

Figure S3. BET specific surface area plot of POM-Pt@COF-TB.

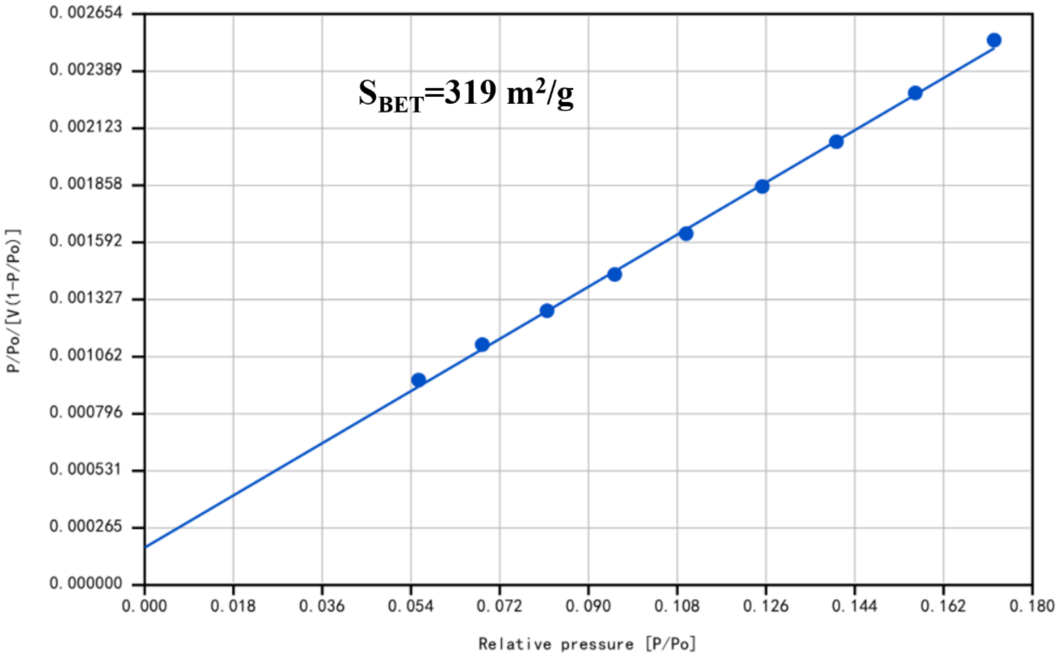

Figure S4. BET specific surface area plot of $Co_{0.75}Fe_{0.25}$@COF-TB.

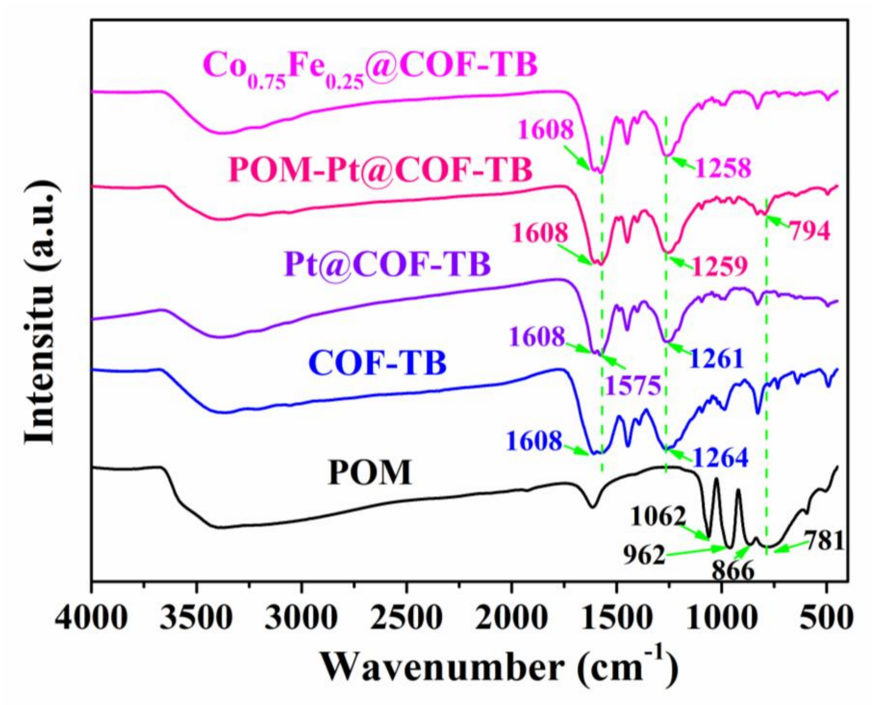

Figure S5. FT-IR spectra of POM, COF-TB, Pt@COF-TB, POM-Pt@COF-TB and $Co_{0.75}Fe_{0.25}$@COF-TB.

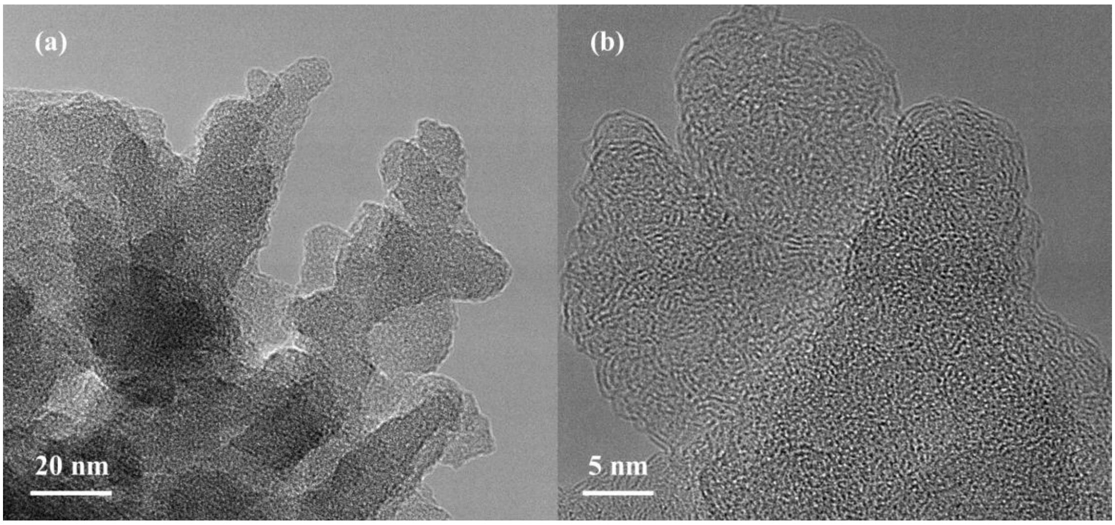

Figure S6. TEM of $Co_{0.75}Fe_{0.25}$@COF-TB.

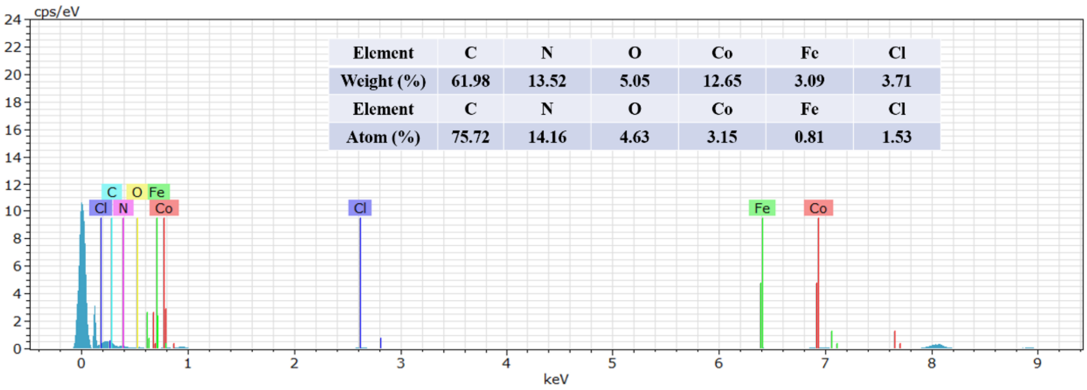

Element	C	N	O	Co	Fe	Cl
Weight (%)	61.98	13.52	5.05	12.65	3.09	3.71
Element	C	N	O	Co	Fe	Cl
Atom (%)	75.72	14.16	4.63	3.15	0.81	1.53

Figure S7. EDS spectra of $Co_{0.75}Fe_{0.25}$@COF-TB.

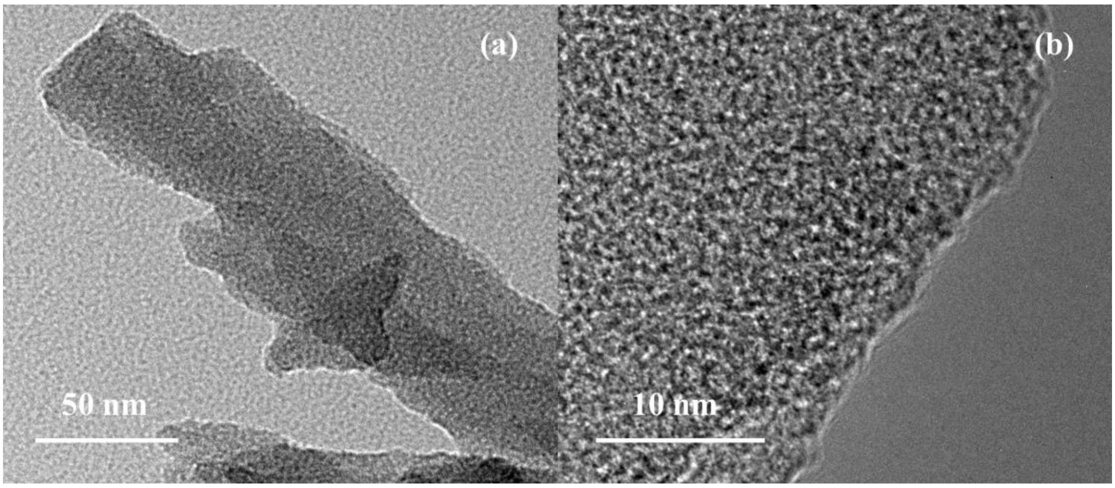

Figure S8. TEM of POM-Pt@COF-TB.

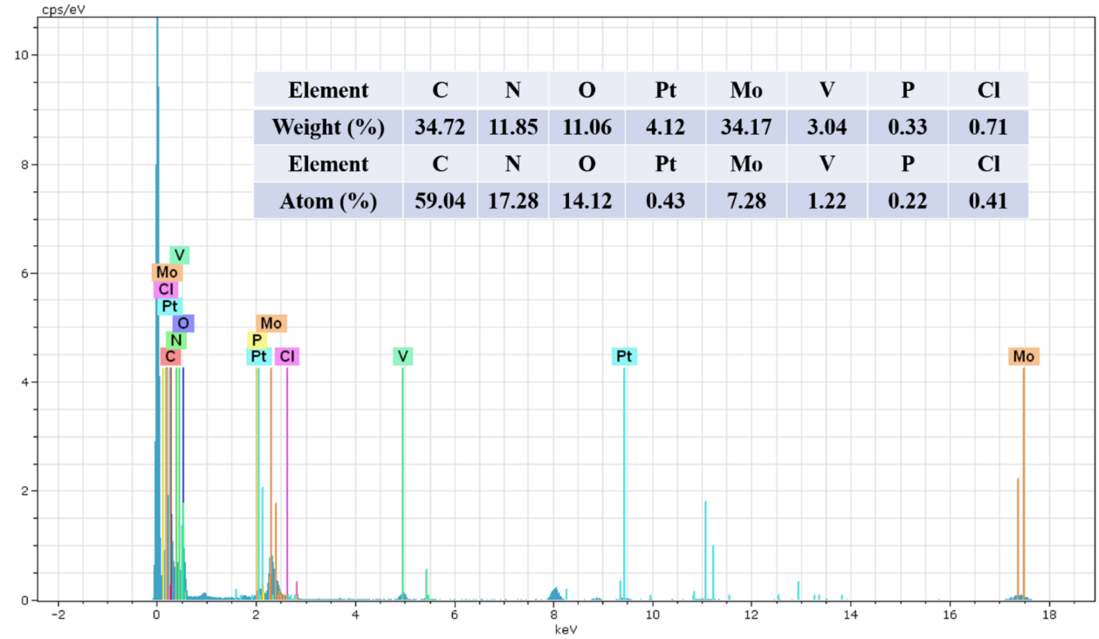

Element	C	N	O	Pt	Mo	V	P	Cl
Weight (%)	34.72	11.85	11.06	4.12	34.17	3.04	0.33	0.71
Element	C	N	O	Pt	Mo	V	P	Cl
Atom (%)	59.04	17.28	14.12	0.43	7.28	1.22	0.22	0.41

Figure S9. EDS spectra of POM-Pt@COF-TB.

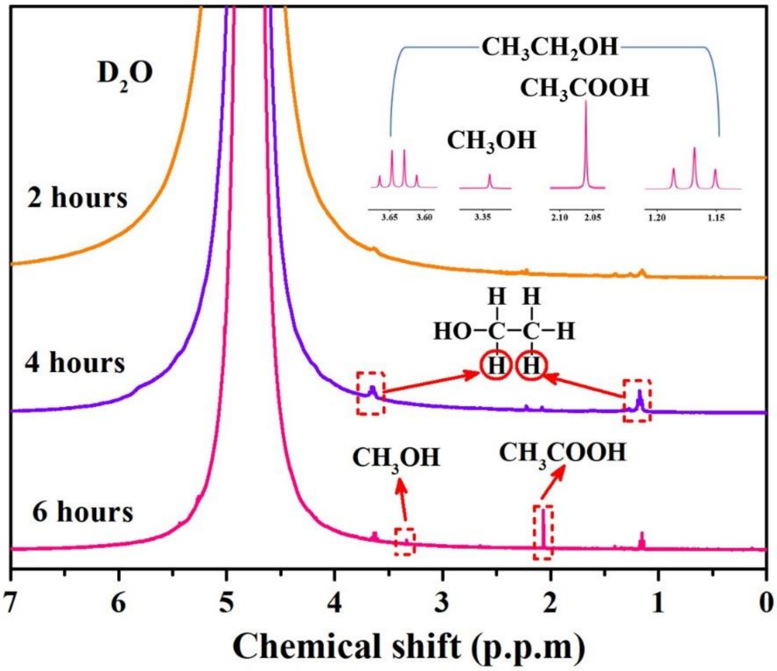

Figure S10. ^{1}H NMR spectra of the methane oxidation reactions products.

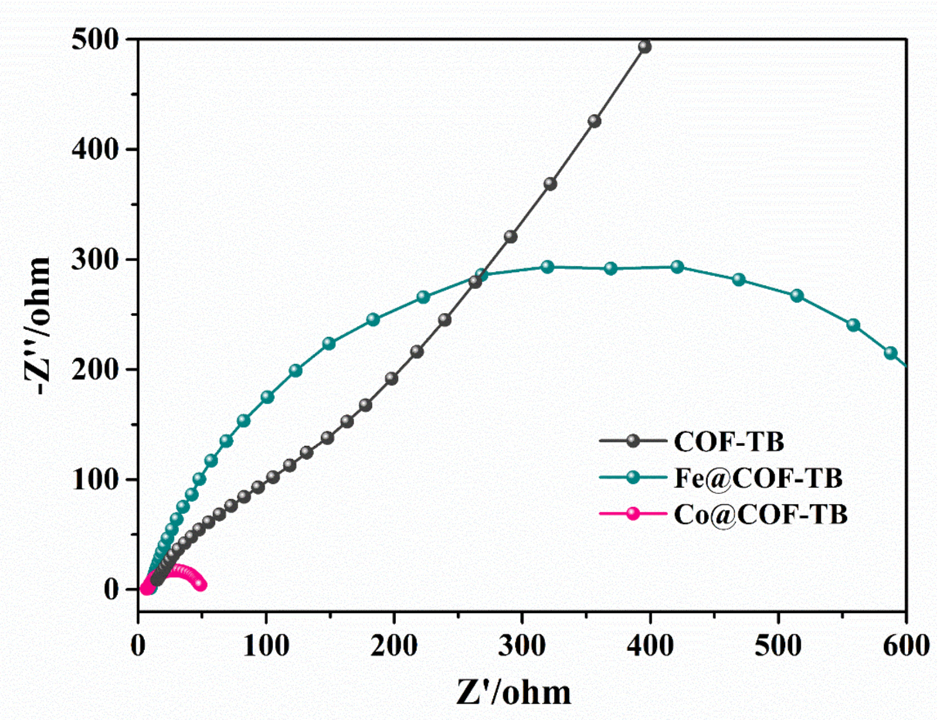

Figure S11. Nyquist plots of COF-TB, Fe@COF-TB and Co@COF-TB.

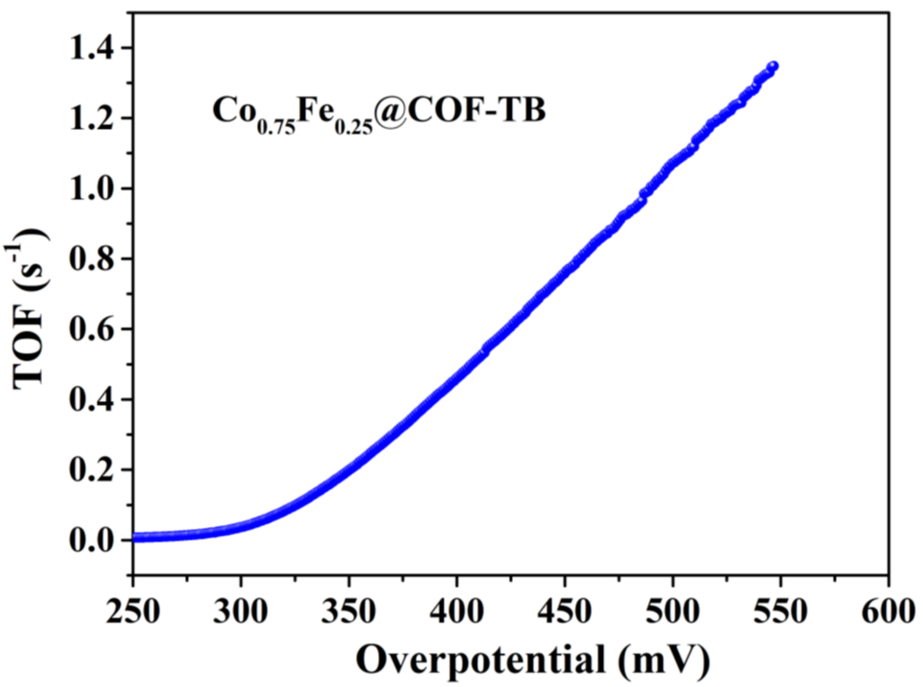

Figure S12. TOF value vs overpotential of $Co_{0.75}Fe_{0.25}$@COF-TB.

Table S1 Comparison of the reported OER catalytic performance of metal-containing COFs catalysts.

Materials	Overpotential	TOF (s^{-1})	Ref
COF-TpDb-TZ-Co	390@10 mA cm^{-2}	0.1@345 mV	S1
Co@COF-Pyr	450@10 mA cm^{-2}	0.1@370 mV	S2
$Co_{0.5}V_{0.5}$@COF-SO_3	318@10 mA cm^{-2}	0.098@300 mV	S3
Co-TpBpy	400@1 mA cm^{-2}	-	S4
$Co_{0.75}Fe_{0.25}$@COF-TB	331@10 mA cm^{-2}	0.119@330mV	This work

References

S1. Liang Y., Xia T., Wu Z., Yang Y., Li Y., Sui Z., Li C., Fan R., Tian X., Chen Q. Tetrazole-functionalized two-dimensional covalent organic frameworks coordinated with metal ions for electrocatalytic oxygen evolution reaction. Mater. Today Chem. 2022, 24, 100777.

S2. Zhao Y., Yang Y., Xia T., Tian H., Li Y., Sui Z., Yuan N., Tian X., Chen Q. Pyrimidine-functionalized covalent organic framework and its cobalt complex as an efficient electrocatalyst for oxygen evolution reaction. ChemSusChem 2021, 14, 4556-4562.

S3. Gao Z., Yu Z., Huang Y., He X., Su X., Xiao L., Yu Y., Huang X., Luo F. Flexible and robust bimetallic covalent organic framework for reversible switching of electrocatalytic oxygen evolution activity. J. Mater. Chem. A 2020, 8, 5907-5912.

S4. Aiyappa H.B., Thote J., Shinde D.B., Banerjee R., Kurungot S. Cobalt-modified covalent organic framework as a robust water oxidation electrocatalyst. Chem. Mater. 2016, 28, 4375-4379.
